# Perfluorocarbon Nanoparticles: Evolution of a Multimodality and Multifunctional Imaging Agent

**DOI:** 10.1155/2014/746574

**Published:** 2014-06-12

**Authors:** Patrick M. Winter

**Affiliations:** Department of Radiology, Cincinnati Children's Hospital, Cincinnati, OH 45229, USA

## Abstract

Perfluorocarbon nanoparticles offer a biologically inert, highly stable, and nontoxic platform that can be specifically designed to accomplish a range of molecular imaging and drug delivery functions in vivo. The particle surface can be decorated with targeting ligands to direct the agent to a variety of biomarkers that are associated with diseases such as cancer, cardiovascular disease, obesity, and thrombosis. The surface can also carry a high payload of imaging agents, ranging from paramagnetic metals for MRI, radionuclides for nuclear imaging, iodine for CT, and florescent tags for histology, allowing high sensitivity mapping of cellular receptors that may be expressed at very low levels in the body. In addition to these diagnostic imaging applications, the particles can be engineered to carry highly potent drugs and specifically deposit them into cell populations that display biosignatures of a variety of diseases. The highly flexible and robust nature of this combined molecular imaging and drug delivery vehicle has been exploited in a variety of animal models to demonstrate its potential impact on the care and treatment of patients suffering from some of the most debilitating diseases.

## 1. Introduction

Perfluorocarbon nanoparticles consist of a liquid perfluorocarbon core encapsulated within a monolayer of phospholipids [1–6]. The particles are around 250 nm in diameter allowing them to circulate easily through capillary beds. To manufacture these particles, the individual components, perfluorocarbon, phospholipids, water, imaging agents, targeting ligands, and drugs, are forced under high pressure through a microfluidizer to form small particles with a fairly narrow size distribution. Perfluorocarbon is biologically inert, highly stable, nontoxic, and not metabolized in the body [7, 8]. The imaging agents and targeting ligands are typically coupled to modified phospholipids allowing for controlled orientation of these compounds, such that they point out into the surrounding biological environment. Nanoparticles can support large payloads of imaging agents, targeting ligands, or drugs due to their large surface area. Incorporating multiple targeting ligands on each particle improves the avidity for the desired biomarker and can reduce the disassociation of the particles from the cell. By anchoring multiple imaging agents on each particle, the detection limit of the contrast agent can be lowered, allowing sensitive localization of biomarkers expressed at very low concentrations.

Perfluorocarbon nanoparticles provide a highly versatile platform that can be modified to serve several different biomedical applications. By attaching targeting ligands onto the particle surface, they can be specifically directed to bind biomarkers of angiogenesis, cancer, thrombosis, or other diseases. Once the particles target a cell population or physiological process associated with a particular disease state, several different options can be realized by further modification of the surface components. Site targeted imaging can be achieved by incorporating imaging agents onto the surface. The perfluorocarbon nanoparticles can be specially formulated for detection by ultrasound [9–12], MRI [2, 13–16], CT [17], optical imaging [18], or nuclear imaging [19, 20]. Since each imaging modality utilizes different contrast agents, it is relatively easy to design nanoparticle formulations that are compatible with multiple modalities by incorporating more than one contrast agent in a single formulation. The strengths and weaknesses of each modality can guide which instrumentation is used for each different application. For example, in vivo imaging of a biomarker expressed at very low concentrations may require a highly sensitive modality, such as nuclear imaging. On the other hand, microscopic analysis of cellular subpopulations within a tumor mass would probably require an optical imaging agent.

The nanoparticles can also be modified to carry a drug payload and specifically deliver it to areas of pathology [21–23]. Lipophilic drugs are the easiest to incorporate within the particle membrane. These drugs disperse within the phospholipid membrane, since they are not soluble in the perfluorocarbon core or in the aqueous environment outside of the particles. Highly lipophilic drugs do not readily disassociate from the particles due to their hydrophobic nature. Instead, the drug is only released from the particle carrier when it comes into close contact with other phospholipid membranes, such as the surface of a targeted cell. The close proximity of the membranes allows the phospholipid components to be exchanged between the particle and the cell, facilitating transfer of the drug to the cell along with these phospholipids. Therefore, when the particles are free in the blood stream, the drug is not released into the tissue. Instead, the drug is only released when the particle binds to the target cell and the two membranes are in contact for a prolonged period of time.

A combined imaging and drug delivery agent can be produced by incorporating an imaging agent on the surface and a drug within the membrane of the perfluorocarbon nanoparticles. The imaging capability provides a means to monitor the particle uptake into the desired tissue. By inference, the amount of drug delivered to the tissue can also be evaluated. This information can be used to verify that adequate drug levels are being delivered to the diseased tissue and also to provide a very early prediction of the subsequent therapeutic effect.

## 2. Targeting Agents

Directing a nanoparticle agent to a specific biomarker relies on incorporating a targeting molecule onto the particle surface. Traditionally, antibodies that bind to cellular receptors have been utilized to direct imaging or drug delivery vehicles to pathological tissues. For example, antibodies that target biomarkers associated with angiogenesis, macrophages, fibrin, inflammation, or tumors have been utilized for molecular imaging or drug delivery applications. Specifically, one of the first demonstrations of molecular imaging with perfluorocarbon nanoparticles utilized anti-fibrin antibodies to image thrombi with ultrasound [24]. Another early study used antibodies that bind the *α*_*ν*_ *β*_3_-integrin to image tumor angiogenesis with MRI [25].

There are a number of limitations with antibodies, however. First of all, antibodies are generally species specific. An antibody developed in mouse studies may not efficiently target the corresponding biomarker in humans. This limitation can be overcome by humanizing the antibody or by using animal models based on human tissues, such as human cancer cells implanted in immunocompromised mice.

Another significant drawback of antibody targeting agents is that they are relatively large, complex, and delicate molecules. The large size of antibodies limits the number of targeting agents that can be incorporated onto the nanoparticle surface. Packing too many antibodies on the surface can degrade the particle stability, produce a steric hindrance to target binding, or interfere with an imaging agent that may also be present on the particle surface. Antibodies are also highly complex molecules, with intricate folding patterns that form a binding region, immune signaling regions, hinge regions, and connecting regions. The complex structure must be considered when designing how the antibody will be coupled to the nanoparticle surface. The coupling scheme cannot alter the binding region or the affinity of the antibody for the target will be degraded. Furthermore, the delicate nature of antibodies limits the chemical processes that can be used during nanoparticle formulation. Temperature extremes, nonphysiological pH values, organic solvents, or other harsh conditions experienced during particle fabrication can denature the antibodies leading to impaired biomarker binding. In particular, perfluorocarbon nanoparticles are generated in a microfluidizer under very high pressures. The high pressure would destroy the antibody structure and ruin the affinity for the biological target. Therefore, antibody targeting agents are typically coupled to the particle surface after the nanoparticles are formulated.

Coupling antibodies to perfluorocarbon nanoparticles can be accomplished with avidin-biotin binding reactions. For example, fibrin targeted particles can be formulated by including biotinylated phospholipids on the surface. For in vitro experiments, a fibrin clot is serially exposed to biotinylated anti-fibrin antibodies, washing steps, avidin, washing, biotinylated particles, and another round of washing [26, 27]. Similar approaches have been used to demonstrate molecular imaging of tissue factor expressed on cultured vascular smooth muscle cells [28, 29]. For in vivo experiments, the biotinylated particles can be mixed simultaneously with avidin and biotinylated antibodies, such as LM609, to target angiogenesis in tumors [25]. However, this approach offers very poor control over how many antibodies couple to each particle and large variations in the consistency of antibody to particle coupling. An alternative approach has been demonstrated for coupling biotinylated anti-Robo4 antibodies to nanoparticles utilizing a phospholipid that binds to avidin [30]. While separate steps are still required to couple avidin to the nanoparticles and then antibodies onto the avidin, this method allows better control over each coupling reaction.

Another common targeting approach utilizes peptides or peptidomimetics coupled to the particle surface. These molecules are much smaller, less complex, and more durable than antibodies. They can be assembled to mimic the binding region of an antibody without all the complex folding and signaling regions. They can be chemically coupled directly to a phospholipid and formulated onto the particle along with the other components. A peptidomimetic that binds to the *α*_*ν*_ *β*_3_-integrin has been used extensively to target perfluorocarbon nanoparticles to angiogenic vasculature associated with cancer [31, 32], cardiovascular disease [33–35], obesity [36], and revascularization [37].

## 3. Imaging Modalities

Perfluorocarbon nanoparticles have been used with a number of different imaging modalities, including ultrasound [12, 28], MRI [26, 31–40], nuclear imaging [19, 20], computed tomography (CT) [17], and florescent imaging. For ultrasound imaging, the perfluorocarbon core itself serves as the contrast generating entity. The speed of sound is very different in biological tissues compared to liquid perfluorocarbon. When the particles bind to the intended target, they induce an impedance mismatch and generate acoustic reflections. The reflected ultrasound waves are detected by the imaging system and produce image contrast.

For CT imaging, a similar approach is utilized. The perfluorocarbon itself has radio-opaque properties and can be detected by CT. Improvements in the CT contrast can be achieved by formulating the particles with other compounds that attenuate the X-ray beam. For example, particles containing iodinated oil in the core improved the CT contrast by a factor of 4.5 [17].

In order to trace perfluorocarbon nanoparticles via nuclear imaging or fluorescent imaging, a tracer must be incorporated onto the particle surface. For nuclear imaging, a radioactive tracer can be coupled to the particles just prior to injection. Single-photon emission computed tomography (SPECT) or positron emission tomography (PET) can subsequently trace the uptake of the particles to tumors, normal tissues, or clearance organs. For fluorescent imaging, an optical imaging dye can be coupled to a phospholipid during particle formulation to anchor the agent onto the particle surface. The dye can be detected in vivo via whole-body optical imaging, or the particle uptake can be investigated with microscopic analysis of tissue slices [41].

**Figure 1:**
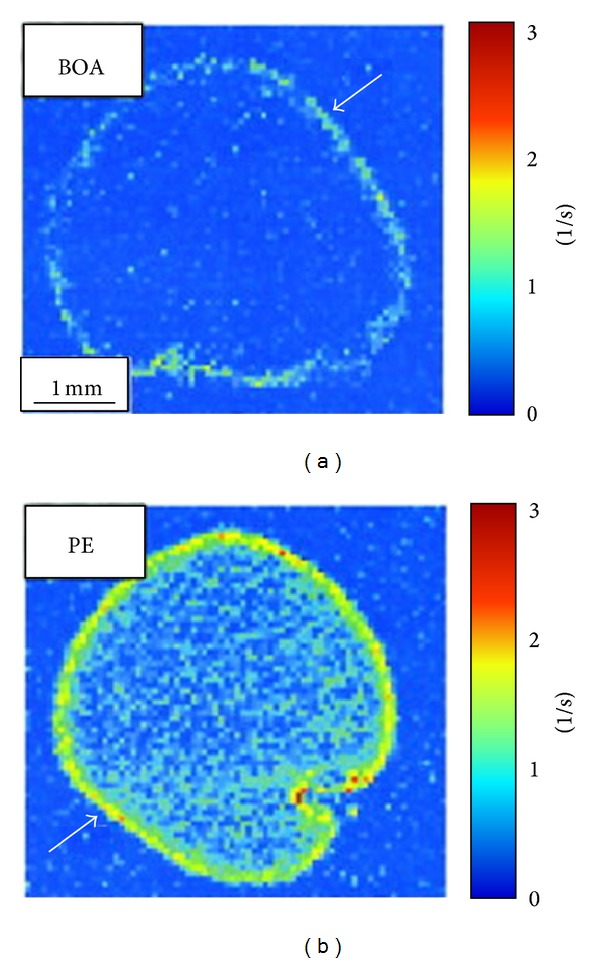
MRI of human plasma clots treated with fibrin targeted perfluorocarbon nanoparticles formulated with either Gd-DTPA-BOA (a) or Gd-DTPA-PE (b) as the imaging contrast agent. The Gd-DTPA-BOA nanoparticles increased the R1 value at the clot surface by 48%, while the Gd-DTPA-PE particles increased the R1 by 72%. The improved relaxation effect was not a result of increased binding of the Gd-DTPA-PE particles to the clot surface, since both agents produced identical gadolinium levels on the clot samples: 0.22 ± 0.01 *μ*mol Gd^3+^ for Gd-DTPA-BOA particles and 0.22 ± 0.02 *μ*mol Gd^3+^ for Gd-DTPA-PE particles. Reprinted with permission from [39].

For MRI, the most common way to generate image contrast with perfluorocarbon nanoparticles is to incorporate paramagnetic chelates onto the particle surface [42]. Gadolinium chelates are the most efficient way to induce MRI contrast in T1-weighted images, and the particle surface can accommodate large numbers of chelates, up to 90,000 per particle [29]. By loading a large payload to gadolinium agents onto each particle, the relaxivity of the total agent increases dramatically [39], up to 2,500,000 (s ∗ mM)^−1^, compared to 4 (s ∗ mM)^−1^ for clinically approved small molecule MRI contrast agents. This extremely high relaxivity is needed in order to detect biomarkers which tend to be expressed at very low levels, such as *μ*M to pM concentrations. Interestingly, the choice of the gadolinium chelating agent can have a dramatic effect on the resulting relaxivity. Particles formulated with a Gd-DTPA-PE agent have significantly higher relaxivity than particles containing Gd-DTPA-BOA [39], leading to a 50% higher paramagnetic effect at the target site (Figure 1). Furthermore, utilizing a DOTA chelate provides a much stronger bond to the gadolinium ion than the DTPA chelate, potentially improving the stability and safety of the contrast agent [43].

In addition to using MRI to detect the gadolinium label on the particle surface, MRI can be used to directly image the fluorine signal arising from the perfluorocarbon core [44–50]. The fluorine signal provides a very high intrinsic signal level, which is only 17% lower than the commonly used proton signal. Another very strong advantage of imaging the fluorine signal from perfluorocarbon particles is that there is no competing naturally occurring fluorine signal in the body. All the fluorine signal detected by MRI arises from the particles themselves, providing a unique and definitive signature of the particles [51–54]. However, there are a number of significant challenges associated with fluorine MRI. First of all, the fluorine signal is often spread over several peaks. This can lead to imaging artifacts and reduces the available signal from each individual peak. In addition, the MRI signal is proportional to the concentration of the tracer. While the intrinsic signal intensity of fluorine is similar to proton, the fluorine concentration in the tissue is many orders of magnitude lower than the proton concentration, resulting in low signal to noise ratio and/or low resolution in fluorine images. Another drawback of fluorine imaging is that dedicated MRI coils and electronics are required to generate radio frequency pulses and record the signal at the fluorine frequency. Research imaging systems can generally be modified relatively easily, but clinical systems are usually much more difficult to reconfigure for fluorine imaging.

One consistent challenge with molecular imaging is that images need to be collected before and after the injection of the contrast agent. The change in signal intensity generated by the nanoparticle agent is generally quite subtle. Careful comparison of the image intensity before and after injection is needed to identify areas of particle binding. One MRI method that does not require pre- and postinjection imaging uses paramagnetic chemical exchange saturation transfer (PARACEST) agents [55].

**Figure 2:**
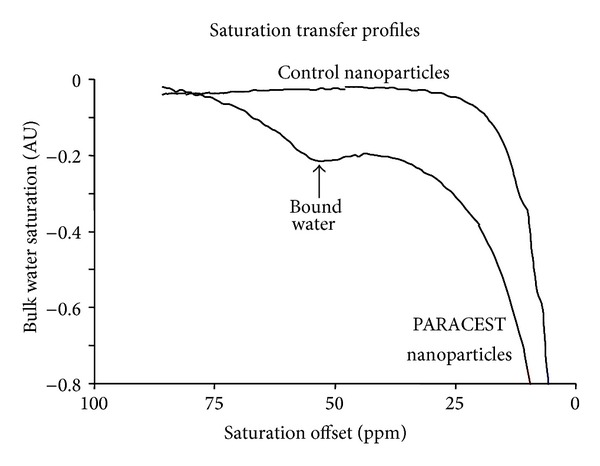
PARACEST nanoparticles display a very obvious saturation transfer effect at 52 ppm (arrow), resulting from saturation of the bound water peak which is effectively transferred into the bulk water pool. Nanoparticles that do not contain the PARACEST chelate do not show any saturation transfer effects at this frequency offset. Reprinted with permission from [56].

Perfluorocarbon nanoparticles formulated with a PARACEST chelate showed a clear saturation transfer effect when a presaturation radiofrequency pulse was applied at 52 ppm (Figure 2) [56]. A pulse at this frequency causes the bound water peak at 52 ppm to be saturated and the water exchange characteristics of the PARACEST chelate allow this saturated magnetization to be transferred into the bulk water pool, producing a lower water signal acquired at 0 ppm. Nanoparticles that lacked the PARACEST chelate did not generate any appreciable saturation transfer at this frequency offset. When targeted to clot samples, the PARACEST particles produced a contrast to noise ratio of 10.

**Figure 3:**
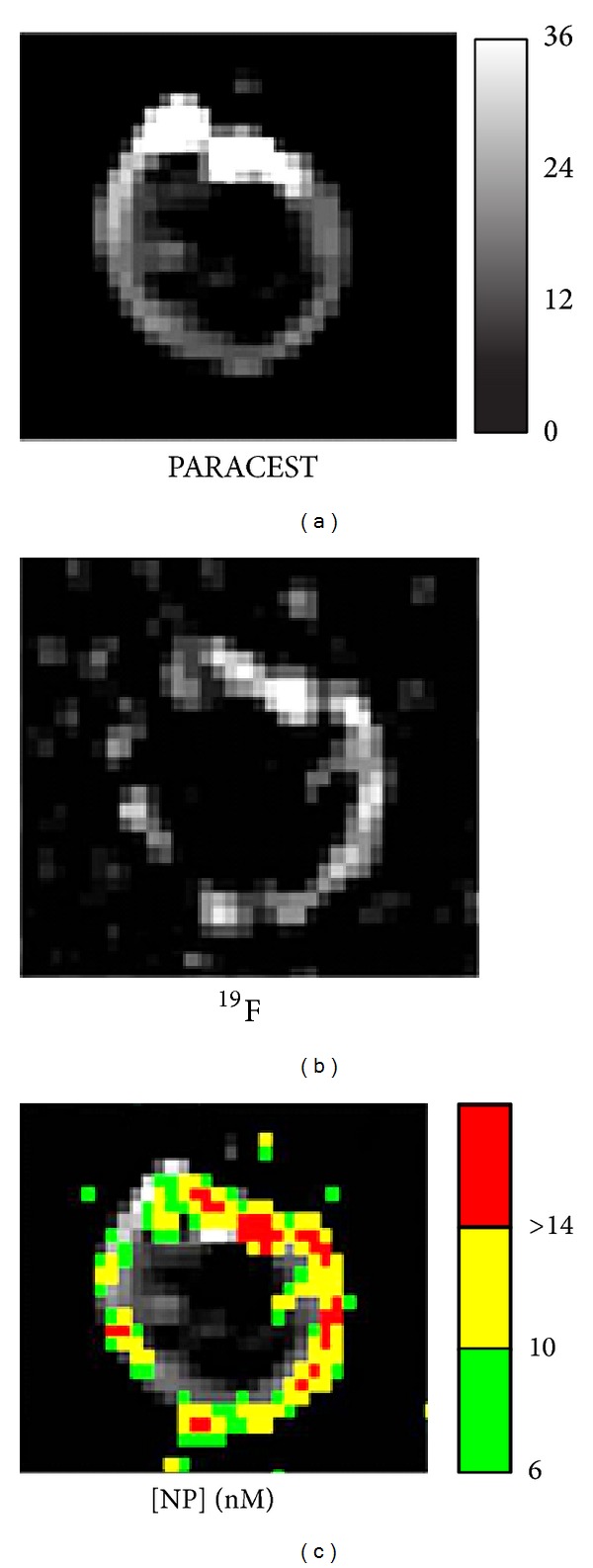
Dual PARACEST and fluorine MRI of fibrin targeted PARACEST perfluorocarbon nanoparticles bound to the surface of a clot. The PARACEST contrast to noise ratio map (a) and fluorine image (b) both show nanoparticles bound to the surface of the clot. The fluorine signal intensity was calibrated to measure the nanoparticle concentration on the clot surface in nM. The particle concentration map is color-coded and overlaid onto the PARACEST subtraction image (c), demonstrating colocalization of these two definitive signals and allowing the water exchange kinetics to be estimated after binding to a biological target. Reprinted with permission from [57].

The contrast generated with PARACEST agents is highly dependent on the water exchange kinetics of the chelate. The kinetics can be modeled mathematically and evaluated in phantom studies. However, measuring the water exchange when the agent is bound to a biological target is not always straightforward because the local concentration of the agent must be known to calculate an accurate value. PARACEST perfluorocarbon nanoparticles enable the measurement of both the PARACEST contrast, through proton MRI, and the particle concentration, through fluorine MRI, allowing the kinetics to be measured on a pixel by pixel basis after the agent has bound to the target (Figure 3) [57]. Mathematical modeling of the PARACEST contrast showed that the optimum bound water lifetime was 970 *μ*s. When the PARACEST particles were diluted in water, the bound water lifetime was 100 *μ*s. When the particles bound to the surface of a clot, the bound water lifetime increased to 600 *μ*s, which is much closer to the optimum value. The increase in the bound water lifetime improved the detection limit of the PARACEST nanoparticles from 4.1 nM (when diluted in water) to 2.3 nM (when bound to the target).

## 4. Molecular Imaging

Angiogenesis is a well-established biomarker associated with most types of solid tumors. As a tumor grows, it needs to establish its own blood supply to provide sufficient oxygen and nutrients to the highly metabolic cells. Tumors typically induce the body to form new blood vessels by secreting vascular endothelial growth factor (VEGF) or other proangiogenic factors. Drugs that block the VEGF signaling of tumors, such as avastin, have proven to be highly effective at inhibiting tumor growth [58, 59]. The *α*_*ν*_ *β*_3_-integrin is an adhesion molecule that becomes upregulated upon initiation of angiogenesis. This integrin is involved in endothelial cell recruitment and proliferation, which are important steps in the formation of new blood vessels.

Perfluorocarbon nanoparticles have been formulated with a peptidomimetic that binds to the *α*_*ν*_ *β*_3_-integrin and a paramagnetic contrast agent for detection by MRI. This agent has been used to image angiogenesis in rabbit tumors [31, 60] and human tumors implanted in mice [61]. In the tumor bearing rabbits, angiogenesis was predominantly located in the tumor rim, where the MRI signal increased by 125% two hours after nanoparticle injection. The image enhancement in the center of the tumor was significantly lower. Histological staining with an antibody that binds to the *α*_*ν*_ *β*_3_-integrin supported the MRI finding that angiogenesis primarily occurred in the tumor periphery. Despite the highly asymmetric and localized distribution of angiogenesis in this animal model, the *α*_*ν*_ *β*_3_-integrin targeted perfluorocarbon nanoparticles were able to reliably and accurately map the expression of this important tumor biomarker in vivo. The large payload of gadolinium ions on the particle surface and the long circulating time of the agent in the blood pool are two crucial features of perfluorocarbon nanoparticles that allow sensitive detection of biological processes associated with pathological tissues.

Similar to the growth of cancerous lesions, angiogenesis is a central feature in the growth and ultimate fate of atherosclerotic plaques [62]. Angiogenesis proliferates from the vasa vasorum to feed the metabolic demands of growing plaques [63]. High levels of angiogenesis are associated with high risk plaques that are prone to rupture, leading to clinical events such as unstable angina, myocardial infarction, and stroke [64–66]. Noninvasive mapping of plaque angiogenesis could be an important metric in determining a patient's cardiovascular risk level or in evaluating the response to a wide variety of therapeutic interventions.

Molecular imaging of plaque angiogenesis was demonstrated with *α*_*ν*_ *β*_3_-integrin targeted paramagnetic nanoparticles and MRI in atherosclerotic rabbits [34, 67]. Cholesterol-fed rabbits exhibit the very early stages of plaque growth in the aorta, including intimal thickening, angiogenesis within the vessel wall, and inflammation. MRI of the aortic wall before and after nanoparticle injection showed an overall signal enhancement of 47 ± 5% [34]. Angiogenesis was very heterogeneous, with individual imaging slices enhancing by as much as 80% and single imaging pixels enhancing by greater than 100%.

Monitoring plaque angiogenesis may also serve as a useful index of therapeutic effect during pharmacological treatments. For example, monitoring the effect of weight loss drugs beyond just the changes in body mass could provide unique insights into the expected cardiovascular outcomes from these treatments. The effect of benfluorex treatment on JCR:LA-cp rats was studied with perfluorocarbon nanoparticles targeted to angiogenesis. The JCR:LA-cp rat is a model of metabolic syndrome displaying obesity, hyperlipidemia, and insulin resistance. In addition, these animals develop spontaneous atherosclerotic plaques and myocardial ischemia [68–71]. The model is a result of a mutation in the leptin receptor, which is often called the corpulent gene (cp) [70]. Homozygous animals (cp/cp) develop obesity and metabolic syndrome. Heterozygous (cp/+) and normal (+/+) animals are indistinguishable and display a lean phenotype. Benfluorex is an anorectic and hypolipidemic drug that decreases insulin resistance, normalizes the lipid profile, and diminishes aortic plaque in JCR:LA-cp rats [72–74].

**Figure 4:**
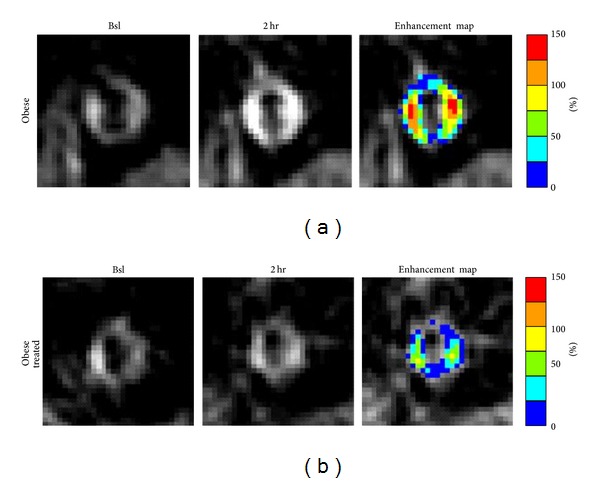
Molecular imaging of aortic angiogenesis with targeted perfluorocarbon nanoparticles targeted to the *α*_*ν*_ *β*_3_-integrin. Images show the abdominal aorta of an obese JCR rat (a) and an obese rat treated with benfluorex (b) at baseline (Bsl) and 2 hours (2 hr) after injection of nanoparticles. High MRI signal enhancement (Enhancement map) was observed in the untreated obese animal, indicating active angiogenesis in the aortic wall to support the development of atherosclerotic lesions. Benfluorex reduced the aortic enhancement in the treated animal, suggesting reduced angiogenesis in the vessel wall. Reprinted with permission from [36].

Treatment with benfluorex caused the obese rats to decrease their weekly food consumption by 30% [36]. After 8 and 16 weeks of benfluorex treatment, the MRI signal enhancement in the aortic wall of obese rats was at least 50% lower than the aortic signal from untreated obese rats (Figure 4). Histological analysis of the aortas from obese rats showed large adventitial adipose deposits. Subintimal plaque thickening was prevalent in the untreated obese rats, but not in the benfluorex-treated animals. Anti-von Willebrand factor staining of endothelial cells revealed that adventitial microvessel density was much higher in the untreated obese rats (5 ± 0.7 vessel/100 *μ*m^2^) compared to the benfluorex-treated obese rats (2.6 ± 0.3 vessel/100 *μ*m^2^) verifying the results obtained with MRI molecular imaging of angiogenesis.

While atherosclerosis is associated with increased angiogenesis in the aortic wall to support plaque formation, the disease also causes reduced angiogenesis in response to tissue ischemia. In particular, peripheral vascular disease is caused by atherosclerotic plaques in the vessels of the legs, which block sufficient blood flow. As an added complication, atherosclerosis inhibits angiogenesis that is needed to reestablish a normal blood supply to the limbs [75, 76]. To evaluate perfluorocarbon nanoparticles as a way to monitor peripheral vascular disease, atherosclerotic rabbits with unilateral ligation of the femoral artery were imaged [37]. A portion of the animals were treated with L-arginine to increase the angiogenic response to the ischemic injury. MRI enhancement in the ischemic limb of the untreated rabbits reached 140% two hours after nanoparticle injection. In the L-arginine treated animals, MRI enhancement reached 250%, confirming successful augmentation of the angiogenic response to ischemia. Both histological and X-ray angiography experiments confirmed the MRI findings. Histological analysis of CD31 expression showed that L-arginine treated rabbits had increased capillary density in the ischemic leg compared to untreated animals. Furthermore, X-ray angiography of the hind limbs showed that untreated rabbits had an angioscore of 0.58, while L-arginine treatment increased the angioscore to 0.85. While this study showed that *α*_*ν*_ *β*_3_-integrin targeted nanoparticles can be used to monitor angiogenesis in skeletal muscle, other potential applications include mapping the angiogenic response to myocardial ischemia or stroke. Furthermore, other angiogenic therapies could be tracked with this contrast agent, such as delivery of growth factors or gene constructs to ischemic tissues.

## 5. Drug Delivery

Perfluorocarbon nanoparticles can be formulated to serve as drug delivery vehicles for a variety of therapeutic agents. For example, paclitaxel and doxorubicin have been incorporated into the particles to inhibit the proliferation of smooth muscle cells [28], a contributing factor to restenosis following angioplasty procedures. Since the drugs are sequestered within the perfluorocarbon nanoparticles, the biodistribution and elimination kinetics are determined by the properties of the particle carrier. As a result, some typical adverse effects, such as doxorubicin cardiotoxicity [77], can be avoided because the particles prevent drug uptake in normal tissues. In another study, rapamycin was locally delivered to the vessel wall with *α*_*ν*_ *β*_3_-integrin targeted nanoparticles after overstretch injury to prevent the development of obstructive plaques [78]. Another drug that has been utilized with perfluorocarbon particles is fumagillin, which blocks angiogenesis. Fumagillin inhibits methionine aminopeptidase 2 (MetAP2) [79, 80] and prevents the proliferation of endothelial cells, with little effect on other cell types [79, 81]. Site targeted delivery of fumagillin has been demonstrated in animal models of cancer and atherosclerosis [82].

In a rabbit Vx-2 tumor model, treatment with *α*_*ν*_ *β*_3_-integrin targeted fumagillin nanoparticles reduced the tumor volume (470 ± 120 mm^3^) compared to untreated animals (980 ± 80 mm^3^, *P* < 0.05) [32]. Without treatment, MRI enhancement was detected in 7.2% of the pixels making up the tumor rim. The tumors treated with targeted fumagillin nanoparticles showed much less angiogenesis in the tumor rim, consisting of only 2.8% of the pixels.

**Figure 5:**
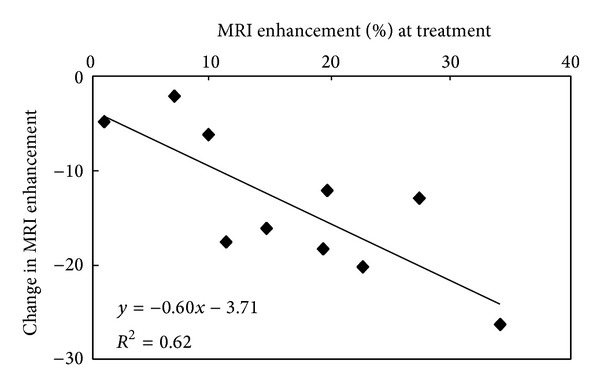
The amount of MRI signal enhancement in the abdominal aorta of atherosclerotic rabbits following treatment with *α*_*ν*_ *β*_3_-integrin targeted fumagillin nanoparticles correlates with the therapeutic effect measured 1 week later. This finding suggests that combined imaging and therapeutic nanoparticles can be used to predict the treatment effects at the time of treatment. Reprinted with permission from [35].

Similar studies have been performed in atherosclerotic rabbits [33, 35]. The aorta of cholesterol-fed rabbits showed an MRI enhancement of 16.7% ± 1.1% after injection with *α*_*ν*_ *β*_3_-integrin targeted fumagillin nanoparticles. Seven days after treatment, the site targeted delivery of fumagillin reduced the MRI enhancement in the aorta to 2.9% ± 1.6%. Histology of the aortic wall showed that fumagillin treatment reduced the number of microvessels by about 50%. The MRI enhancement at the time of treatment was compared with the change in enhancement observed 1 week after treatment to evaluate if MRI could predict the subsequent therapeutic response. The signal at the time of therapy showed good correlation with the treatment response: *R* ^2^ = 0.62 (Figure 5). These results suggest that perfluorocarbon nanoparticles with combined imaging and therapeutic functionalities can be used to confirm and quantify the local delivery of chemotherapeutics, as well as to provide an early prognosis of the anticipated treatment effects.

## 6. Control Experiments

Although *α*_*ν*_ *β*_3_-integrin targeted nanoparticles have been utilized for molecular imaging and drug delivery applications, appropriate control experiments must also be performed to clearly assess the effectiveness of the targeting methods. For molecular imaging of tumor angiogenesis, the MRI enhancement obtained with *α*_*ν*_ *β*_3_-integrin targeted paramagnetic nanoparticles was compared with several control formulations [31]. One control experiment used nanoparticles lacking the targeting ligand, which produced only about half as much image enhancement as the targeted agent. Another control experiment employed competitive blockage of the targeted imaging agent. Rabbits were treated with *α*_*ν*_ *β*_3_-integrin targeted nanoparticles that did not contain the imaging agent, followed by injection of the targeted paramagnetic nanoparticles. The nonparamagnetic particles occupied the *α*_*ν*_ *β*_3_-integrin binding sites and blocked the uptake and retention of the targeted paramagnetic agent, resulting in lower MRI signal enhancement in this group of animals.

In addition to using control nanoparticle formulations, the MRI enhancement arising from control tissues can be compared to the signal from the tumor. One control tissue is nearby muscle, which showed only very low levels of signal enhancement after nanoparticle injection. Another control tissue was somewhat unexpected because it appeared to be a tumor on the MRI scans, but it was actually inflammation resulting from a rejected tumor implantation. The mass did not show any signal enhancement after injection of *α*_*ν*_ *β*_3_-integrin targeted nanoparticles and subsequent histological analysis showed no angiogenesis in the mass.

Similar control experiments were conducted in the atherosclerotic rabbit study [34]. Nontargeted paramagnetic nanoparticles and competitive blockade with targeted nonparamagnetic nanoparticles produced about half as much MRI enhancement compared to the targeted imaging agent. In addition, nonatherosclerotic rabbits were imaged, which showed significantly less enhancement than the diseased animals.

The experiments in JCR rats [36] and the rabbit model of peripheral vascular disease [37] utilized drug treatments, benfluorex and L-arginine, to modulate the amount of angiogenesis in these animals. In these drug studies, untreated animals served as controls. In addition, normal control littermates were compared to JCR obese animals to evaluate the effect of obesity on angiogenesis in the aortic wall. In the peripheral vascular disease rabbits, the femoral artery in one hind limb was ligated, leaving the other limb to serve as an internal control. In untreated animals, the MRI enhancement in the ischemic limb was 49% higher than in the control limb, while the difference was 104% in L-arginine treated animals.

The targeted antiangiogenesis treatment study in rabbit tumors with fumagillin loaded nanoparticles utilized a number of control nanoparticle formulations [32]. The treated animals received *α*_*ν*_ *β*_3_-integrin targeted fumagillin nanoparticles. Control groups received nontargeted fumagillin nanoparticles, *α*_*ν*_ *β*_3_-integrin targeted nanoparticles without drug, or saline. All three control treatments resulted in larger tumors and more angiogenesis compared to the *α*_*ν*_ *β*_3_-integrin targeted fumagillin group. In a similar way, the targeted fumagillin treatment study in atherosclerotic rabbits used control treatments consisting of nontargeted fumagillin nanoparticles, *α*_*ν*_ *β*_3_-integrin targeted nanoparticles without drug, or saline [35]. All three control treatments resulted in higher residual angiogenesis compared to the *α*_*ν*_ *β*_3_-integrin targeted fumagillin group.

## 7. Conclusions

Perfluorocarbon nanoparticles have proven to be a highly flexible and reliable platform for multimodality molecular imaging and multifunctional drug delivery in a wide variety of biomedical applications. The primary advantages of this agent are (1) prolonged residence in the blood stream which allows multiple passes through the target tissue increasing the probability of binding to the biomarker of interest, (2) a large surface area which can support high payloads of imaging agents and targeting molecules in order to lower the detection threshold of the imaging modality and increase the avidity for the desired target, (3) selective delivery of therapeutic agents only during close contact between the target cell membrane and the particle surface to reduce drug uptake in collateral tissues, (4) compatibility with a range of medical imaging modalities allowing rational selection of the preferred instrumentation based on the strengths and weaknesses of each method, and (5) opportunity to incorporate a variety of targeting ligands that could be based on antibodies, peptides, peptidomimetics, or other molecular structures that bind to biomarkers associated with an extensive assortment of diseases or normal biological processes.

While the applications of perfluorocarbon nanoparticles reported here have mostly focused on molecular imaging of angiogenesis, alternative formulations could be developed for other biomarkers, such as inflammation, apoptosis, hypoxia, and cancer cell receptors. In addition to these molecular imaging and drug delivery examples, this agent has been explored for cell labeling [47], siRNA transfection [83], in vivo delivery of cytolytic compounds [84, 85], measuring blood oxygen tension [86], dissolving thrombi [87], and other medical applications. With a diverse portfolio of diagnostic and therapeutic functions, perfluorocarbon nanoparticles have a promising role in many of the cutting edge technologies that are currently being developed to fight the most pressing challenges facing the field of medicine today.
